# Search for Key Genes and Functional Pathways of Ulcerative Colitis to Colon Cancer Based on Bioinformatics

**DOI:** 10.3389/fonc.2022.857148

**Published:** 2022-03-15

**Authors:** Shengbao Wang, Lingling Zhen, Xiaoli Li, Xu Fu, Peiwu Li, Dekui Zhang

**Affiliations:** ^1^ Emergency Center, Gansu Emergency Medicine Clinical Research Center，Lanzhou University Second Hospital, Lanzhou, China; ^2^ Infectious Department, Lanzhou University Second Hospital, Lanzhou, China; ^3^ Digestive Department, Lanzhou University Second Hospital, Lanzhou, China; ^4^ Key Laboratory of Emergency Medicine, Lanzhou University Second Hospital, Lanzhou, China; ^5^ Department of Gastroenterology, Key Laboratory of Digestive Diseases Lanzhou University Second Hospital, Lanzhou, China

**Keywords:** ulcerative colitis, colon cancer, key genes, diffuse inflammatory disease, cancer

## Abstract

Ulcerative colitis (UC) is a persistent and diffuse inflammatory disease of the intestine. It is widely prevalent in developed countries. Approximately 30% of patients with UC suffer from widespread and aggressive colitis and are at increased risk of colon cancer. In this study, the genetic features and potential molecular mechanisms shared between UC and colorectal cancer were investigated. The datasets from GEO and TCGA were analyzed to obtain differentially expressed genes, of which there were 116 overlapping genes. A module containing 15 genes was obtained using String and Cytoscape to analyze the module and identify hub genes. Weighted gene co-expression network analysis (WGCNA) was used to identify co-expression modules associated with UC and colon cancer, with 52 overlapping genes. Functional clustering of the two gene cohorts was performed using the Metascape online tool, with three significant functions or pathways associated with both gene cohorts. A total of 19 key genes were included, and CCT2 was identified after expression and survival analyses. CCT2 is highly expressed in colon cancer and lowly expressed in UC, and its low expression is associated with a poor prognostic ratio. This study reveals, for the first time, that CCT2 may be a promoter of UC transformation into colon cancer and identifies new gene candidates that could be used as biomarkers or potential therapeutic targets.

## Introduction

Ulcerative colitis (UC), a type of inflammatory bowel disease (IBD), is a persistent and diffuse inflammatory disease with typical symptoms, such as bloody diarrhea, abdominal pain, and stool urgency (1). UC is a chronic immune-mediated disease that is widely prevalent in developed countries and is rapidly emerging in newly industrialized countries (2). Some data have shown that its prevalence is about 0.286% in the United States, 5.05% in Norway, and 0.00667% in Malaysia (3). UC is common in all age groups but highly prevalent in adults over 6050 years of age (4). Family genetic susceptibility and previous smoking history are among the most important risk factors associated with UC. UC is clinically heterogeneous, and approximately 30% of patients suffer from a widespread and aggressive form of colitis (5). Since there is no cure, patients usually seek pharmacological treatment. However, the inability to achieve durable remission leads approximately 15% of patients to opt for surgical removal of most or even all of the colon within 20 years of diagnosis (6).

Persistent inflammatory irritation of the colon puts patients with UC at a significantly increased risk of developing colon cancer, and the leading cause of death in patients with UC is colitis-associated cancer (CACC) (7). Although several studies have been conducted in this area, the causes and mechanisms responsible for this transformation are currently not well understood. This may be due to the glycosylation of the oncoprotein MUC1, which undergoes abnormal changes in UC, and this abnormally glycosylated MUC1 promotes the development and progression of cancer (8). For example, a study by Michael Kvorjak et al. proposed that immune cells may affect the abnormal glycosylation of MUC1, which induces cancer. In addition, Yao et al. started with inflammation and elaborated that validation and inflammatory cytokines may promote the progression of UC and colitis-related cancers (9). These studies attempt to find the factors that jointly promote the progression of UC and colon cancer, and hopefully, this will induce a therapeutic entry point.

Microarray and bioinformatics approaches have been widely used to screen for genetic changes at the genomic level (10). In this study, mRNA microarray datasets from the gene expression omnibus (GEO) and the cancer genome atlas (TCGA) were analyzed to obtain differentially expressed genes in UC and colon cancer tissues. Protein–protein interaction (PPI) network analysis was then performed to explore the relationship between the different genes. The weighted gene co-expression network was also used to obtain key genes associated with UC and colon cancer. The functional clustering of key genes was analyzed jointly with the Metascape online tool to find the functional pathways linking UC and colon cancer progression. Finally, the key genes associated with the progression of both diseases were obtained by analyzing the gene expression levels and survival analysis.

## Methodology

### Data Sources

Microarrays for UC were obtained from the GEO (https://www.ncbi.nlm.nih.gov/geo/) database. GSE37283 included four pure UC samples, five normal samples, and 11 UC samples with tumor formation. GSE38713 included 13 normal samples and 30 UC samples. GSE74604 included 30 normal samples and 30 colorectal cancer samples. Gene expression matrix and clinical information data of colon cancer and paracancer were obtained from the TCGA database, including 41 normal samples and 456 tumor samples. GSE37283, GSE38713, and GSE74604 were integrated into a new dataset containing normal, UC, and COAD tissues named Merge_1.

### Variance Analysis

The samples were divided into normal, UC, and tumor tissues. The datasets were analyzed separately for variance analysis using the R package limma package. |log2FC| > 1 and P < 0.05 were defined as screening thresholds. Upset plots are a visualization tool for showing the common or unique differential genes between GSE37283, GSE38713, and TCGA.

### PPI Network Construction and Module Analysis

PPI networks were constructed using the String online database to analyze the functional interactions between proteins. The MCODE plug-in in Cytoscape was used to cluster the generated networks, defining tightly connected regions into a module. The selection criteria for the MCODE plug-in were Degree Cutoff = 2, Node Score Cutoff = 0.2, K-Core = 2, Max. Depth = 100. 0.2, K-Core = 2, and Max. Depth = 100.

### Weighted Gene Co-Expression Network Analysis (WGCNA)

WGCNA can be used to identify highly synergistic gene sets, and identify candidate biomarker genes or therapeutic targets according to the interconnection of gene sets and the association between gene sets and phenotypes. WGCNA was used to find biologically significant co-expressed gene modules and to explore the relationship between gene networks and diseases. In this study, WGCNA was used to obtain gene modules associated with UC and colon cancer. First, a suitable soft threshold β was selected according to the criteria of a scale-free network. Then, a hierarchical clustering dendrogram was constructed, and similarly expressed genes were classified into different modules. Finally, appropriate gene modules were selected by calculating the correlation between module feature genes (ME) and clinical features.

### Functional Clustering of Gene Modules

Metascape is a web-based portal that provides comprehensive gene list annotation and analysis resources (11). Metascape was used to simultaneously analyze key genes from PPI and WGCNA for pathway enrichment and functional annotation of genes to deeply understand the association between genes and diseases. Thus, the common or unique functions and pathways of the genes in both modules were annotated. The key gene sets were enriched and analyzed to understand their Kyoto gene Encyclopedia (KEGG) pathway and Gene Ontology (GO).

### Expression of Key Genes

The GSE37283, GSE38713, and GSE74604 datasets were merged using the R package named GM1. The merged datasets contained normal, UC, and colon cancer tissue samples, and differences in the expression of key genes were observed using a one-way ANOVA. Survival analysis of key genes was performed using the R package SURVIVAL, and a log-rank test was used to analyze the survival differences between the high and low expression of genes.

## Results

### Identification and Analysis of Differentially Expressed Genes (DEGs)

**Figure 1:**
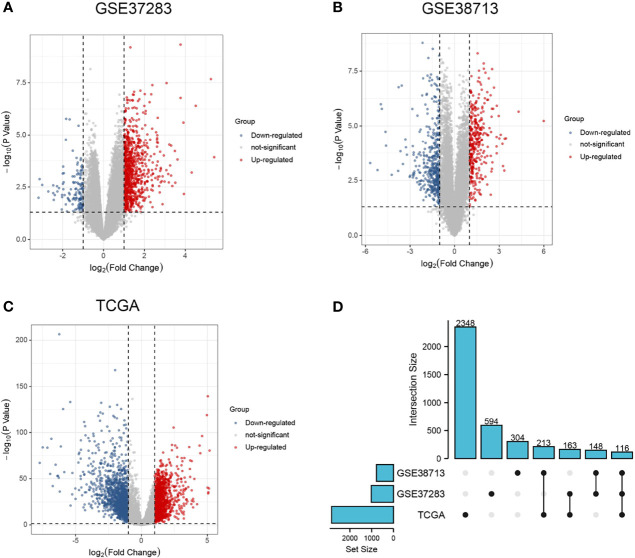
Differential expression analysis and Upset plots. **(A–C)** are the differential analysis from GSE37283, GSE38713, and TCGA datasets, respectively, and the results are shown as volcano plots. Blue indicates downregulation, and red indicates upregulation. **(D)** Upset plots showing the intersection or concatenation parts of the three differentially expressed genes.

DEGs were identified in the GSE37283, GSE38713, and TCGA datasets, respectively. In GSE37283, there were 134 downregulated genes and 887 upregulated genes (**Figure 1A**). In GSE38713, 372 downregulated genes and 330 upregulated genes were found (**Figure 1B**). There were 1439 downregulated genes and 1401 upregulated genes in the TCGA–COAD genes (**Figure 1C**). As shown in the Upset plot, the common and unique parts of the three groups of DEGs were shown, in which a total of 116 genes overlapped (**Figure 1D**).

### Analysis of Key Modules of DEGs

**Figure 2:**
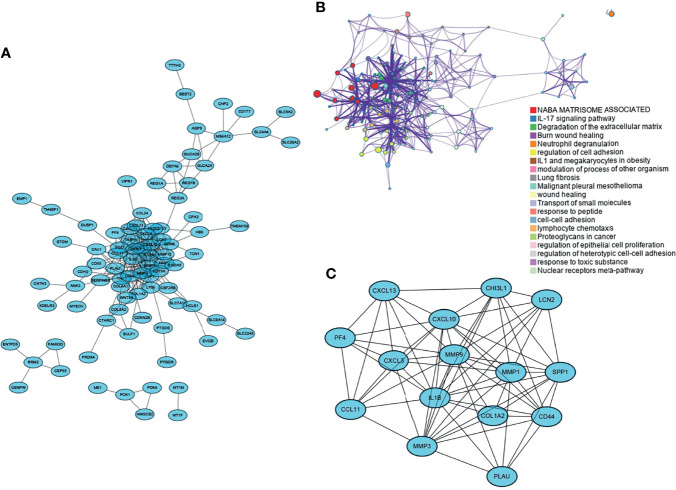
Functional interoperability network of DEGs and selection of key modules. **(A)** PPI network of DEGs; **(B)** rich terminology network in DEGs, each color represents one most prominent term; **(C)** most important gene modules in the PPI network.

The screened DEGs were imported into the STRING database for PPI network construction and visualized using Cytoscape (**Figure 2A**). Networks with rich terms in DEGs were visualized, and the top 20 most significant terms included NABA MATRISOME ASSOCIATED, the IL-17 signaling pathway, and other functions or pathways (**Figure 2B**). Six gene modules were obtained using the MCODE plug-in in Cytoscape, and the most tightly connected module was selected. It included 15 nodes and 69 edges (**Figure 2C**).

### Selection of Co-Expression Modules for UC and COAD

**Figure 3:**
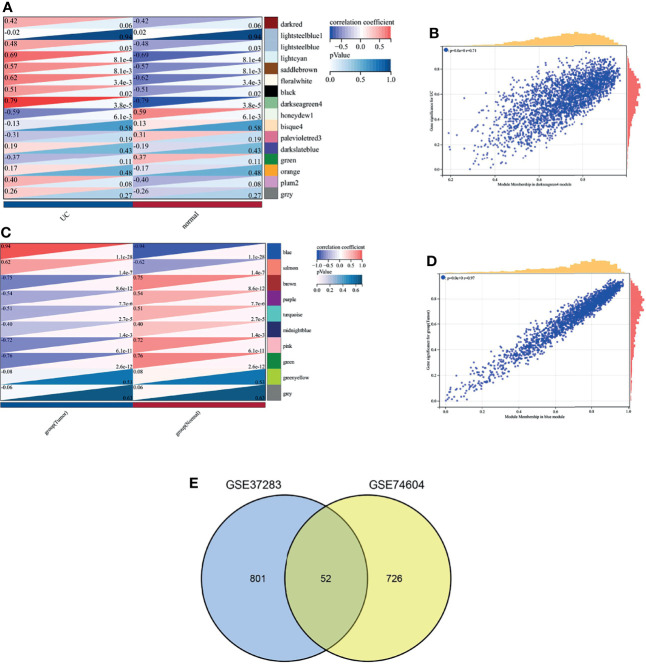
Weighted gene co-expression network analysis. **(A)** Heat map of correlation between gene modules and phenotypes in GSEA37283; **(B)** Scatter plot of correlation between key module genes and phenotypes in GSEA37283; **(C)** Heat map of correlation between gene modules and phenotypes in GSE74604; **(D)** Scatter plot of correlation between key module genes and phenotypes in GSEA74604; **(E)** Venn diagram of two sets of key module genes.

Sixteen and 10 modules were identified by WGCNA in GSE37283 and GSE74604, respectively, with each color representing a different module. Heat maps of module–phenotype correlations were drawn based on Spearman correlation coefficients to assess the relationship between modules and features (**Figures 3A, C**). In GSE37283, darkseagreen4, floral white, and light cyan were highly correlated with UC, and darkseagreen4 (R = 0.79, P = 3.8e−5) was selected as the key module (**Figure 3B**). In GSE74604, blue salmon was highly associated with COAD, and blue (R = 0.94, P = 1.1e−28) was selected as the key module (**Figure 3D**). The intersection of the genes in the two key modules was taken using a Venn diagram, and 52 overlapping genes were obtained (**Figure 3E**).

### Functional Clustering

**Figure 4:**
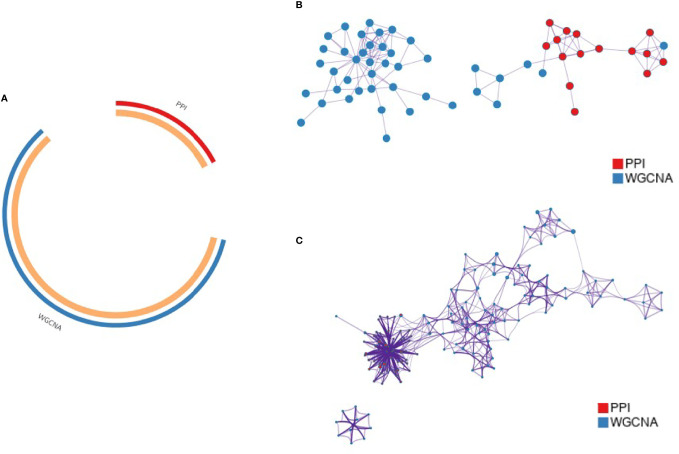
Functional clustering of key genes by Metascape. **(A)** Circos plot showing the overlap of key gene modules from PPI and WGCNA; **(B)** PPI network of genes in the merged cohort; **(C)** Pie chart showing the enriched terminology network of genes, with different colors indicating different cohorts.

This study was conducted using Metascape to understand the functions and pathways of key genes obtained using different analysis methods. The key gene modules obtained using PPI and WGCNA analyses were named PPI-KG and WGCNA-KG, respectively. The overlap and shared term connections between them are shown as circle plots (**Figure 4A**). The two sets of gene lists were merged for PPI network analysis, and the functional terms between them were colored separately according to the counts (**Figure 4B**). A subset of enriched terms was selected and presented as a network graph to further capture the relationships between terms, where terms with a similarity > 0.3 were connected by edges (**Figure 4C**). The top 20 clusters and their representative enriched terms are presented in the table, with the regulation of neutrophil degranulation, ossification, and protein hydrolysis being the pathways significantly enriched in both gene lists, containing a total of 19 genes (**Table 1**).

**Table 1:** Top 20 clusters and their representative enrichment terms.

_PATTERN_	Category	Description	Count	Log10(P)
PPI	WikiPathways	Overview of proinflammatory and profibrotic mediators	10	–20.38
PPI	KEGG Pathway	IL-17 signaling pathway	8	–16.39
WGCNA	GO Biological Processes	ribonucleoprotein complex biogenesis	12	–10.97
PPI	WikiPathways	Lung fibrosis	5	–10
PPI	Canonical Pathways	PID AVB3 OPN PATHWAY	4	–8.91
WGCNA	WikiPathways	Nucleotide metabolism	4	–7.53
PPI	Reactome Gene Sets	Cell surface interactions at the vascular wall	4	–6.27
PPI WGCNA	Reactome Gene Sets	Neutrophil degranulation	9	–5.95
PPI WGCNA	GO Biological Processes	ossification	7	–5.62
WGCNA	GO Biological Processes	nucleobase-containing compound biosynthetic process	9	–5.47
WGCNA	GO Biological Processes	protein import into mitochondrial matrix	3	–5.27
PPI	KEGG Pathway	Amoebiasis	3	–4.78
WGCNA	GO Biological Processes	positive regulation of phosphatase activity	3	–4.48
WGCNA	Reactome Gene Sets	ESR-mediated signaling	5	–3.86
WGCNA	GO Biological Processes	regulation of translation	6	–3.85
WGCNA	Reactome Gene Sets	Translation	5	–3.84
WGCNA	GO Biological Processes	translational initiation	3	–3.75
PPI WGCNA	GO Biological Processes	regulation of proteolysis	8	–3.63
PPI	GO Biological Processes	regulation of peptidyl-serine phosphorylation	4	–3.52
PPI	GO Biological Processes	regulation of chromosome organization	4	–3.3

### Enrichment Analysis

**Figure 5:**
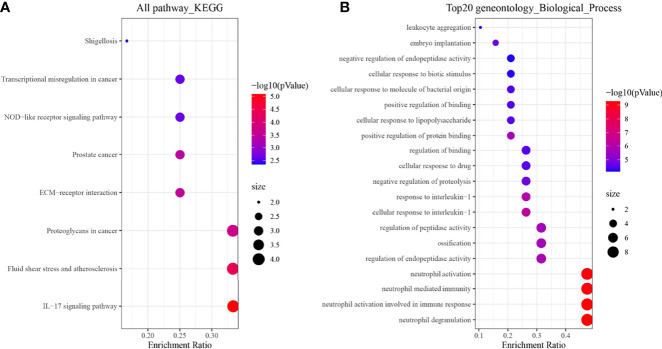
Enrichment analysis results of 19 genes. **(A)** KEGG; **(B)** Biological process terms of the top 20 GO.

Based on the above results, we enriched and analyzed 19 genes in an attempt to understand their biological pathways or functions. The results showed that 19 genes were most associated with the KEGG pathway of IL − 17 signaling pathway (**Figure 5A**); In biological processes, it is related to neutrophil activation, degranulation, and its mediated immune response (**Figure 5B**).

### Expression and Survival Analysis

**Figure 6:**
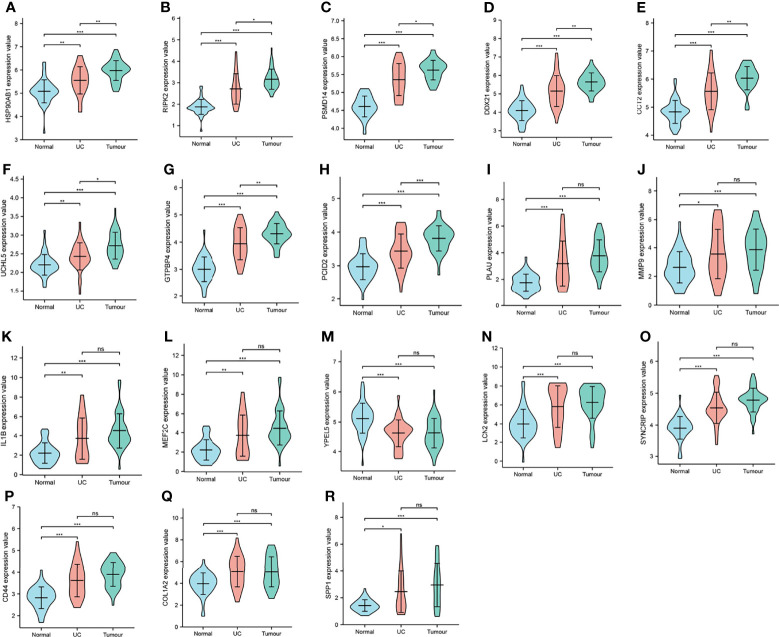
Expression distribution of key genes. **(A–R)** Expression level distribution of 18 key genes in normal, UC, and COAD tissues. *P < 0.05, **P < 0.01,***P < 0.001, ns, no significant. CHIL1 was not significantly different in all the three tissues, so it is not shown.

**Figure 7:**
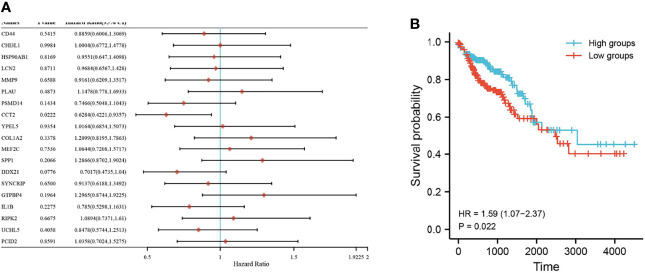
Survival of key genes in colorectal cancer samples. **(A)** Forest plot with one-way Cox analysis for 19 genes; **(B)** Survival curve plot for CCT2. P < 0.05 is considered statistically significant.

Nineteen genes related to the regulatory pathways of neutrophil degranulation, ossification, and protein hydrolysis were extracted and analyzed for their expression levels in different tissues in Merge_1. Most genes were significantly differentially expressed in normal and COAD tissues or in normal and UC tissues (**Figure 6**). Only HSP90AB1, RIPK2, DDX21, UCHL5, GTPBP4, and PCID2 were significantly different in UC and COAD tissues, and they all showed overexpression in colon cancer tissues compared to UC tissues (**Figures 6A–H**). A univariate survival analysis of 19 genes revealed that only CCT2 was associated with colon cancer prognosis, and its low expression was associated with poor prognosis (**Figures 7A, B**).

## Discussion

In recent years, the cellular and molecular mechanisms by which chronic inflammation is associated with tumorigenesis have been progressively explored. In comparison, patients with a long course of UC have a significantly higher chance of developing colitis-associated cancer (12). This may be due to the chronic inflammation caused by persistent irritation or infection that can induce repeated epithelial cell stimulation and infiltration of immune cells and soluble mediators, thus providing a beneficial environment for tumor development (13, 14). It has also been shown that increased expression of certain proteins or activation of pathways may play an important role in transforming UC into colon cancer (9). In this study, we conducted bioinformatics analysis based on the latter to seek key genes and mechanisms that may promote the transition from UC to colon cancer. Three independent gene microarray datasets and one TCGA-COAD dataset were bioinformatically analyzed. gse37283, gse38713, and TCGA-COAD were used to perform differential analysis, and 116 overlapping DEGs were identified. A key module containing 15 genes was obtained using Cytoscape’s MCODE plug-in. Key modules associated with UC and colon cancer were also identified using WGCNA analysis, with 52 genes. Functional clustering was performed using Metascape for both gene cohorts, which shared functions or pathways for regulating neutrophil degranulation, ossification, and protein hydrolysis.

Neutrophils are the first responders to inflammation and infection, and their elevated ratio to lymphocytes is a prognostic indicator of overall survival in some cancers (15). As an important component of cancer-associated inflammation, neutrophils are recruited in the tumor microenvironment to promote tumor progression (16). While neutrophil degranulation allows the antimicrobial agents contained in the granules to kill bacteria, excessive degranulation can also damage the host tissue (17). Ossification is defined as the formation of bone or bone material, or the process of converting fibrous tissue or cartilage into bone or bone material (18). In contrast, regulating protein hydrolysis is an essential process for all cells, and it is particularly important when cells adapt to a new environment or coordinate development (19). Genes related to the regulation of neutrophil degranulation, ossification, and protein hydrolysis were extracted, and their expression in different tissues and their prognoses concerning colon cancer were observed. Significant differences were obtained in the expression of CCT2 in UC and colon cancer tissues. CCT2 is a subunit of CCT, which is a molecular chaperone protein that contributes to the correct folding of proteins, thus ensuring the stability of dynamic cellular homeostasis (20). CCT expression has been reported to be upregulated in rapidly proliferating tumor cells to promote tumor growth through efficient protein production (21). CCT expression has been shown to drive cell invasion and proliferation (22), and elevated mRNA levels of most of its subunits are associated with poor tumor prognosis (23). As one of the important subunits of CCT, CCT also plays an important role in tumors. Studies have shown that CCT2 expression negatively correlates with survival in breast cancer patients, which supports our results (24). CCT2 can also regulate protein folding functions associated with hypoxia in colorectal cancer (25). In this study, CCT2 expression showed an increasing trend in normal, UC, and colon cancer tissues. This expression difference also responds to the possibility that CCT2 may be closely associated with the development of colonic inflammation or cancer.

However, it has not been reported whether CCT directly relates to UC transformation into colorectal cancer. The available studies have suggested that CCT may contribute to malignant transformation in an inflammatory setting. The NF-κB pathway has long been recognized as a classic proinflammatory signaling pathway (26). Increased NF-κB activity indirectly promotes the formation of neutrophil extracellular traps (NET), which is one of the antimicrobial mechanisms of neutrophils (27). As with degranulation, the occurrence of this mechanism may allow neutrophils to rapidly enter into suicidal cell death, inducing chronic inflammation and cancer (28). However, CCT may promote NF-κB activity. There is evidence that the binding of LOX-1, a novel CCT substrate protein with an important link between chronic inflammation and cancer (29), to CCT may activate the inflammatory transcription factor NF-κB, thereby inducing malignant transformation (30). Altering the expression level or activity of CCT may also lead to increased NF-κB activity and carcinogenesis (31). This shows that CCT has a proinflammatory role and may also indirectly promote the transformation of inflammation into cancer.

## Data Availability Statement

The original contributions presented in the study are included in the article/supplementary material. Further inquiries can be directed to the corresponding authors.

## Author Contributions

All authors listed have made a substantial, direct, and intellectual contribution to the work and approved it for publication.

## Funding

This work was supported by The Natural Science Foundation of Gansu Province(20JR10RA724), The Second Hospital of Lanzhou University Cuiying Science and Technology Innovation Program Project (CY2020-MS06) and Science and Technology Plan Project of Lanzhou City (2020-ZD-91).

## Conflict of Interest

The authors declare that the research was conducted in the absence of any commercial or financial relationships that could be construed as a potential conflict of interest.

## Publisher’s Note

All claims expressed in this article are solely those of the authors and do not necessarily represent those of their affiliated organizations, or those of the publisher, the editors and the reviewers. Any product that may be evaluated in this article, or claim that may be made by its manufacturer, is not guaranteed or endorsed by the publisher.
